# Epidemiology of acute flaccid myelitis in children in the Netherlands, 2014 to 2019

**DOI:** 10.2807/1560-7917.ES.2022.27.42.2200157

**Published:** 2022-10-20

**Authors:** Jelte Helfferich, Marit MA de Lange, Kimberley SM Benschop, Bart C Jacobs, Coretta C Van Leer-Buter, Adam Meijer, Dewi P Bakker, Eva de Bie, Hilde MH Braakman, Rick Brandsma, Rinze F Neuteboom, Erik H Niks, Jikke-Mien Niermeijer, Vincent Roelfsema, Niels Schoenmaker, Lilian T Sie, Hubert G Niesters, Oebele F Brouwer, Margreet JM te Wierik

**Affiliations:** 1 Department of Neurology, University Medical Center Groningen, University of Groningen, Groningen, the Netherlands; 2 Centre for Infectious Disease Control (CIb), National Institute for Public Health and the Environment (RIVM), Bilthoven, the Netherlands; 3 Department of Neurology and Immunology, Erasmus MC, University Medical Center Rotterdam, Rotterdam, the Netherlands; 4 Department of Medical Microbiology and Infection Prevention, University of Groningen, University Medical Center Groningen, Groningen, the Netherlands; 5 Department of Paediatric Neurology, Amsterdam University Medical Center, Amsterdam, the Netherlands; 6 Department of Paediatric Neurology, Amalia Children’s Hospital, Radboud University Medical Center, Nijmegen, the Netherlands; 7 Department of Paediatric Neurology, University Medical Center Utrecht, Utrecht, the Netherlands; 8 Department of Neurology, Erasmus MC, University Medical Center Rotterdam, Rotterdam, the Netherlands; 9 Department of Neurology, Leiden University Medical Center, Leiden, the Netherlands; 10 Department of Neurology, Elisabeth-Tweesteden Hospital, Tilburg, the Netherlands; 11 Department of Paediatrics, Martini Hospital, Groningen, the Netherlands; 12 Department of Neurology, Isala Hospital, Zwolle, the Netherlands; 13 Department of Paediatric Neurology, Haga Hospital, the Hague, the Netherlands

**Keywords:** Acute flaccid myelitis, acute flaccid paralysis, enterovirus D68, enterovirus A71, surveillance, epidemiology, children

## Abstract

**Background:**

Acute flaccid myelitis (AFM) is a polio-like condition affecting mainly children and involving the central nervous system (CNS). AFM has been associated with different non-polio-enteroviruses (EVs), in particular EV-D68 and EV-A71. Reliable incidence rates in European countries are not available.

**Aim:**

To report AFM incidence in children in the Netherlands and its occurrence relative to EV-D68 and EV-A71 detections.

**Methods:**

In 10 Dutch hospitals, we reviewed electronic health records of patients diagnosed with a clinical syndrome including limb weakness and/or CNS infection and who were < 18 years old when symptoms started. After excluding those with a clear alternative diagnosis to AFM, those without weakness, and removing duplicate records, only patients diagnosed in January 2014–December 2019 were retained and further classified according to current diagnostic criteria. Incidence rates were based on definite and probable AFM cases. Cases’ occurrences during the study period were co-examined with laboratory-surveillance detections of EV-D68 and EV-A71.

**Results:**

Among 143 patients included, eight were classified as definite and three as probable AFM. AFM mean incidence rate was 0.06/100,000 children/year (95% CI: −0.03 to 0.14). All patient samples were negative for EV-A71. Of respiratory samples in seven patients, five were EV-D68 positive. AFM cases clustered in periods with increased EV-D68 and EV-A71 detections.

**Conclusions:**

AFM is rare in children in the Netherlands. The temporal coincidence of EV-D68 circulation and AFM and the detection of this virus in several cases’ samples support its association with AFM. Increased AFM awareness among clinicians, adequate diagnostics and case registration matter to monitor the incidence.

**Key public health message**

**What did you want to address in this study?**

Acute flaccid myelitis (AFM) is a polio-like disease, that mainly affects children. The incidence of AFM in European countries is largely unknown. We aimed to investigate the incidence in the Netherlands and to examine if this coincides with enterovirus detections in the country.

**What have we learnt from this study?**

AFM is a rare disease with few cases diagnosed in children in the Netherlands. The temporal coincidence of occurrence of patients with AFM and enterovirus D68 circulation, as well as the detection of this particular virus in samples from several cases support its association with AFM.

**What are the implications of your findings for public health?**

While the incidence of AFM is low, the impact of this disease on individual patients is often great with persistent disability in many. Therefore, to monitor the incidence of AFM, it is important to insure that awareness of this disease among clinicians increases and persists, that diagnostics are adequate and that cases are registered.

## Introduction

Acute flaccid myelitis (AFM) is a polio-like condition, mainly occurring in children, and characterised by an acute onset of flaccid limb weakness, combined with abnormalities in the grey matter of the spinal cord on magnetic resonance imaging (MRI) and pleocytosis in cerebrospinal fluid (CSF). Weakness can be severe and persistent, leading to significant disability in affected patients [1]. AFM has been associated with different enteroviruses (EV), in particular EV-D68 and EV-A71, with increasing evidence for causality [2-5].

In North America, a biennial upsurge of AFM cases has been reported from 2014 onwards coinciding with an increased detection rate of EV-D68 [6-8]. Unlike in the United States (US), where surveillance targets the clinical picture of AFM, surveillance in Europe up to 2020 has mainly been centred on associated EVs [9]. There, an increased detection rate of EV-D68 occurred in 2014, 2016, 2018 and to a lesser extent in 2019, but the incidence of AFM in these years is not known [10-12]. During these times however, cases and case series of AFM were identified in particular in 2016, when a European working group composed of virologists and clinicians from 20 European countries described 29 EV-D68-positive AFM patients [13]. Also in 2016, an outbreak of EV-A71 occurred in Spain, during which 133 cases of severe neurological disorders were reported, including 12 presenting with a clinical picture compatible with AFM [14].

Despite more focus on EV in Europe, some publications from the United Kingdom have related outcomes from monitoring the clinical syndrome [15,16]. In 2018, coinciding with an increase of EV-D68 detections in this country, 40 cases of acute flaccid paralysis (AFP), defined as an acute onset of limb weakness and flaccidity, were reported. Sixteen cases were further classified as probable or confirmed AFM and for two of them EV-D68 was detected [16].

In North America, in the largest AFM cohort (n = 159) described so far [7], EVs were only detected in 20–45% of cases. This may be related to incomplete or inadequate testing or to testing which nevertheless resulted negative for the virus at the moment weakness occurred [6-8]. Because in AFM cases, EVs are often not detected or tested for, the mainly used ‘EV-focused’ approach in Europe has undoubtedly led to under-reporting of the number of AFM cases.

To be able to estimate the public health impact of AFM and to decide about the necessity and usefulness of introducing AFM surveillance, reliable incidence numbers are crucial. In this study, we aimed to retrospectively identify cases of AFM in the Netherlands and examine the incidence in the context of circulation of EV-D68 and EV-A71.

## Methods

### Identification of acute flaccid myelitis cases

**Figure 1:**
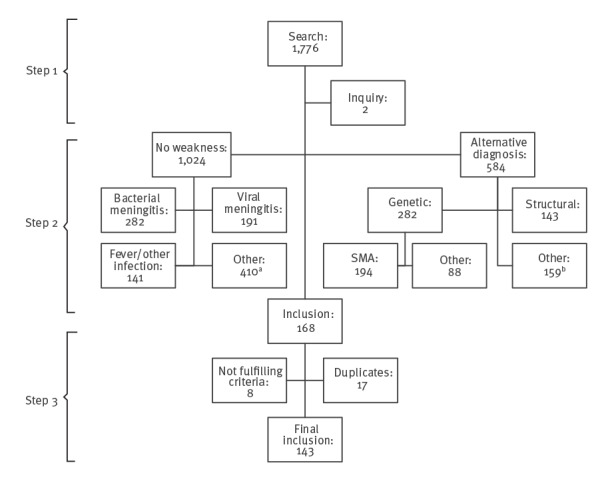
Results of the search and selection procedures, the Netherlands, January 2014–December 2019 (n = 1,778 patients screened)

CMV: cytomegalovirus; SMA: spinal muscular atrophy.

^a^ Other diagnoses in patients without limb weakness included, among others, (suspected) autoimmune encephalitis (n = 57), congenital CMV infection (n = 39) and post-infectious ataxia (n = 29).

^b^ Other diagnoses included, among others, neuroborreliosis (n = 19), slowly progressive spastic paraparesis (n = 12) and chronic inflammatory demyelinating polyneuropathy (n = 10).

A stepwise approach was used to identify cases of AFM (Figure 1). First, electronic health records were searched, for children with disease onset before the age of 18 years and with specific diagnostic codes (International Classification of Diseases (ICD) and Dutch classification of diagnoses ‘Diagnose Behandel Combinatie’ (DBC)), including infectious diseases which affect the nervous system and/or disorders presenting with limb weakness (specific codes are listed in the Supplementary Material). This search was done in 10 Dutch hospitals (six university hospitals and four large community hospitals), covering all, but one, hospitals in the country, with a paediatric neurology department. This way, we presumed to be able to pick up any child presenting with acute weakness in the Netherlands, except for those in the referral area of one hospital. To be included in the search, a diagnostic code had to be registered from January 2014 through December 2019. Additionally, the paediatric neurology staff of every participating hospital was asked to list any cases of suspected AFM apart from the search and selection procedure. That was done to be able to include any suspected AFM cases who might have been missed by using the diagnostic codes, as there was no specific AFM-code in the study period.

In step 1, electronic healthcare systems were searched for diagnostic codes suggestive of disorders presenting with limb weakness and/or infectious diseases affecting the nervous system. Inquiry with the paediatric neurology staff of the participating hospitals yielded two additional cases. In step 2, cases without weakness or with a clear alternative diagnosis were excluded. In step 3, cases diagnosed outside the inclusion period (not fulfilling inclusion criteria) and duplicates/triplicates were excluded.

Second, the files of patients included after the first step were screened. Patients without clinical weakness or with an obvious diagnosis other than AFM were excluded. In cases without weakness and an alternative diagnosis, the absence of weakness was noted as the reason for exclusion.

In case of uncertainty on inclusion, cases were discussed in the research group (minimally comprising JH, OFB, MtW, MdL) to decide on in- or exclusion.

Third, records were checked to exclude any that did not fulfil the January 2014–December 2019 inclusion-period criterion and a structured scoring list was applied to the remaining selected cases. This scoring list covered initial diagnosis, demographic features, clinical characteristics, disease course, and results of ancillary investigations including CSF analysis, MRI, electromyography and nerve conduction studies (EMG), serum analysis for autoantibodies (myelin oligodendrocyte (MOG) and aquaporin 4 (AQP4)), and virological tests. The type and number of samples tested with PCR, as well as positivity for EV-D68, EV-A71 or any other EV in any of these samples was considered in the scoring.

Recently published diagnostic criteria for AFM, proposed by the international AFM working group, were applied to the obtained data, by JH, with two amendments [1]. First, the criterion of decreased muscle tone in at least one weak limb was omitted as this was not included in the scoring list. Second, the presence of demyelinating features on EMG was added as a factor compatible with an alternative diagnosis. Based on these criteria, cases were classified as: (i) definite AFM; (ii) probable AFM; (iii) possible AFM; (iv) uncertain; and (v) alternative diagnosis more likely.

Cases with uncertainty on classification were presented to two experienced clinicians (OFB and BCJ) for reassessment. Both were not aware of the initial classification. The final classification was determined by consensus between these clinicians.

### Calculation of incidence rates

Mean AFM incidence rates over the 6-year period as well as yearly incidence rates, both with 95 per cent confidence intervals (95% CI), were calculated based on the number of cases classified as probable and definite AFM. Population numbers from Statistics the Netherlands (CBS) were used as a reference for the annual number of children under 18 years in the Netherlands. Since one university hospital did not participate in this study, the estimated number of children in their referral region, based on data from CBS, was subtracted from the total number of children in the Netherlands. The referral region as well as the area covered by the other university hospitals are shown in Supplementary Figure S1, where the former and latter are represented on a map of the country in different colours. The number of children in the referral region as well as the total number of children under 18 years of age in the Netherlands are shown in Supplementary Table S1.

### Enterovirus surveillance

Data on the number of EV-D68 and EV-A71 detections (2014–2019) were obtained from two non-overlapping surveillance systems: (i) the general practitioner (GP)-based sentinel influenza like illness (ILI) and other acute respiratory infections (ARI) surveillance (Nivel and RIVM) [17,18], and (ii) the national EV surveillance reported in EV-surveillance/VIRO-TypeNed [19-21].

The GP surveillance is performed year-round by ca 40 practices spread throughout the Netherlands, covering close to 1% of the general population with the percentage of the general population under the age of 20 years also close to 1% [22]. All combined nose and throat specimens collected from patients with ILI or ARI are subjected to RT-PCR for EV and all EV-positive specimens are typed by sequencing [17,18]. During the study period, among the tested patients, the percentage below the age of 18 years was per year on average 25% (range: 21–29%).

The national EV-surveillance comprises a year-round typing of samples from EV positive cases diagnosed in ca 30 medical microbiology laboratories conducting EV diagnostics in the Netherlands and predominantly covers patients attending hospitals with or without hospitalisation. The primary aim of this surveillance system is to exclude the circulation of poliovirus among EV positive cases, and secondary to characterise the circulation of non-polio EVs, such as EV-D68 and EV-A71. Denominator data for the national EV-surveillance system are not available.

## Results

### Search and selection

In a first step, the diagnosis-based search in the electronic health records produced a total of 1,776 patients. Inquiry with paediatric neurology staff of the participating hospitals yielded two additional cases of suspected AFM, who had received a different DBC/ICD-code (DBC 0131 ‘peripheral nerve’ and DBC 0599 ‘other central nervous system conditions’). Screening of the 1,778 patient files in the second step resulted in the exclusion of 1,024 patients without weakness, and 584 with a clear alternative diagnosis, leaving 168 patients eligible for step 3 (Figure 1). From these 168 patients, eight were excluded because they had not been diagnosed in the inclusion period. Fifteen patients were counted twice, and one patient three times due to referrals between hospitals. After exclusion of these cases, 143 patients qualified for further analysis.

### Classification

The diagnostic criteria for AFM, as proposed by the international AFM working group, were applied to the included patients (n = 143), resulting in the classification as shown in Table 1. Fifteen cases were discussed with OFB and BCJ before final classification could be made. In two patients, insufficient information was available for classification (not included in Table 1). Furthermore, after discussion in the consensus meeting, one patient was classified as ‘definite AFM’, while not fulfilling all AFM criteria. This patient had clinical signs and symptoms compatible with AFM, with severe asymmetric flaccid limb weakness and minimal recovery over time. PCR in respiratory material was positive for EV-D68. EMG findings of absent or decreased motor responses in affected muscles, without signs of demyelination, were compatible with AFM. However, MRI of the spinal cord 1 day and 1 week after onset of weakness, both of which were reassessed during the classification process, did not show abnormalities.

**Table 1:** Characteristics of the different classification subgroups in the study, the Netherlands, January 2014–December 2019 (n = 141**^a^**)

Characteristics	Definite^b^ (total = 8)^c^	Probable^b^ (total = 3)	Possible^b^ (total = 3)	Uncertain^b^ (total = 11)	Alternative diagnosis more likely^b^ (total = 116)
Male: female^d^ (percentage)	4:4 (NA^e^)	2:1 (NA^e^)	1:2 (NA^e^)	8:3 (NA^e^)	66:50 (57%)
Median age in years at diagnosis (IQR, full range)	5 (2.3–7.8; 1–11)	12 (NA^e^; 3–15)	2 (NA^e^; 0–15)	5 (3–15.5; 1–16)	6 (3–13; 0–17)
**AFM criteria; proportions of patients**					
Onset to nadir < 10 days	8/8	3/3	3/3	11/11	90/105
Prodrome	6/8	2/3	3/3	11/11	81/114
Hyporeflexia	8/8	3/3	0/3	11/11	91/114
MRI spinal cord abnormalities	7/8	3/3	3/3	0/1^c^	30/61
Predominant grey matter involvement	7/8	3/3	3/3	0/1	21/60
Pleocytosis	8/8	0/3	1/2	0/8	26/106
**Factors suggestive of an alternative diagnosis; proportions of patients**					
Encephalopathy	1/8	0/3	0/3	0/11	14/115
Sensory deficits	2/7	1/3	2/2	0/9	60/93
MRI brain abnormalities	2/6	0/3	0/3	0/4	16/65
Supratentorial white matter/cortex	0/6	0/3	0/3	0/4	15/64
AQP4 antibodies	0/6	0/1	0/2	0/0	0/32
MOG antibodies	1/6	0/1	0/3	0/1	5/26
Demyelination on EMG	0/2	0/1	0/0	0/1	33/54
**Virology; proportions of patients**					
**Sample type investigated**					
Respiratory sample	5/8	2/3	1/3	4/10	29/106
Faecal sample	4/6	1/3	0/3	1/10	22/104
CSF sample	8/8	3/3	1/3	5/10	56/105
All samples tested	3/6	1/3	0/3	1/10	10/105
**Virus detected**					
Enterovirus^f^	5/8	1/3	0/2	0/8	3/72
EV-D68	4/8	1/3	0/2	0/8	0/72
EV-A71	0/8	0/3	0/2	0/8	0/72

AFM: acute flaccid myelitis; AQP4: aquaporin-4; EMG: electromyography including nerve conduction studies; EV-D68: enterovirus-D68; EV-A71: enterovirus-A71; IQR: interquartile range; MOG: myelin oligodendrocyte glycoprotein; MRI: magnetic resonance imaging; NA: not applicable.

^a^ Of 143 patients included in the study, two do not figure in Table 1 because information for classification was insufficient.

^b^ Information on several characteristics was not available for all patients. The number of patients without information are removed from the nominators and denominators of the fractions.

^c^ Including one case without MRI abnormalities but with a compatible clinical picture and positive for EV-D68.

^d^ Information on sex was collected as a binary variable.

^e^ NA, as the numbers are low, so IQRs or percentages are not presented.

^f^ Total number of enteroviruses detected, including EV-D68, EV-A71, other subtypes and untyped enteroviruses. The denominator indicates the number of patients in whom at least one sample was investigated.

Both ‘positive’ AFM criteria and factors that might suggest an alternative diagnosis are shown.

*Definite AFM.* In these eight patients, median age at onset was 5 years (interquartile range (IQR): 2.3–7.8; full range: 1–11). In five patients a respiratory sample was taken (day 2–5 after onset of weakness), four of whom were positive for EV, all subtyped as EV-D68. A faecal sample was taken in four patients (day 2–11 after onset of weakness), one of which was positive for EV, but could not be further subtyped. In the three patients for whom respiratory, faecal and CSF samples were tested, two respiratory samples were positive for EV-D68. In none of the samples, EV-A71 was detected. One patient tested positive for MOG antibodies but was still included in this group after careful consideration, because of lack of sensory abnormalities and significant proximal weakness at follow-up. Of the patients classified as definite AFM, four had also been initially diagnosed as AFM, and four as transverse myelitis.

*Probable AFM.* Of the three patients, one had been diagnosed as AFM and two as transverse myelitis. In two patients, respiratory specimen were tested (both taken at day 2 after onset of weakness), of which one tested positive for EV, subtyped as EV-D68. In the patient with a positive respiratory sample, the faecal sample (day 2 after onset of weakness) and CSF sample were negative.

*Possible AFM.* All three patients were initially diagnosed with transverse myelitis (Table 1). In one of these patients a respiratory and CSF sample were taken, in which no virus was isolated.

*Uncertain diagnosis.* In this group of 11 patients, one was initially diagnosed as AFM, with a compatible clinical picture. In this patient, IgM for EVs in blood was positive, but PCR testing of both respiratory material and faeces was negative. Both initial and repeated MRI of the spinal cord were of suboptimal quality but did not reveal clear abnormalities. All other patients in this group had initially been diagnosed with a Guillain–Barré syndrome (GBS) variant without sensory abnormalities (pure motor GBS).

*Alternative diagnosis.* This group of 116 patients included, among others, 75 patients who had been diagnosed as GBS, 20 as transverse myelitis and eight as acute disseminated encephalomyelitis (ADEM). In three patients an EV was isolated, two in respiratory material, with an EV which could not be further specified, and one in faeces, subtyped as EV CV-A9. The proportion of cases with respiratory or faecal samples in this group was 29/106 (27%) and 22/104 (21%), respectively, which is lower compared with the other groups. In the 10 patients (10%) in whom respiratory, faecal and CSF samples were tested, one respiratory sample was positive for an untyped EV. All CSF samples in both this group and other groups tested negative for EV and other viruses.

### Incidence rate

All the patients classified as probable or definite AFM were seen in university hospitals, often after referral from a community hospital (Supplementary Figure S1). The incidence rate of AFM in the Netherlands, based on cases classified as probable or definite AFM together, was calculated as 0.06/100,000 children/year from January 2014 through December 2019 (95% CI: −0.03 to 0.14). There was variation over the different years with a minimal incidence rate of 0/100,000 children in 2015 and 2017 and a maximum incidence rate of 0.12/100,000 (95% CI: 0.00 to 0.24) children in 2016 (Supplementary Table S1; Yearly incidence rates of probable and definite acute AFM).

### Enterovirus detection

**Figure 2:**
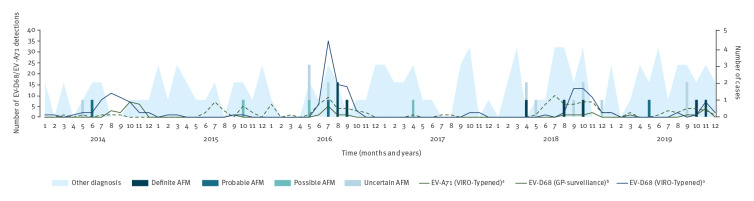
Temporal distribution of number of cases of AFM, according to their classification (bars) and cases with an alternative diagnosis (plane), and monthly number of EV-D68 and EV-A71 detections (lines), the Netherlands, January 2014–December 2019

AFM: acute flaccid myelitis; EV-A71: enterovirus A71; EV-D68: enterovirus D68; GP: general practitioner.

^a^ EV-D68 and EV-A71 detections by enterovirus surveillance/VIRO-TypeNed.

^b^ EV-D68 detections by the GP-surveillance.

Over the 6-year period (2014–2019), 220 EV-D68 positive cases were reported in the two surveillance systems (39 through GP surveillance and 181 through EV-surveillance/VIRO-Typened). Respiratory samples were the main sample type found to be positive for EV-D68. Figure 2 shows that the virus was frequently detected in both surveillance systems in 2014, 2016, 2018, and 2019 as previously reported [12,17,18,23] with clearly high circulation in the autumn and winter months (September through November) in most years. The number of detections in 2019 was low and more spread out across the autumn and winter season, with most detections in December.

EV-A71 was detected only twice in the GP-surveillance system and is therefore not shown.

EV-A71 was detected 130 times through EV-surveillance with similar circulation patterns to those seen in EV-D68 (Figure 2). Only two detections of EV-A71 were made by GP-surveillance during the study period.

A temporal relationship between AFM and EV-D68/A-71 can be suggested from Figure 2, with cases classified as definite or probable most commonly being seen in periods of increased EV-D68/EV-A71 circulation. AFM cases, including EV-D68 positive cases, are, however, also observed before onset of periods with increased EV circulation (Supplementary Table S2; Onset month of EV-positive cases according to definite, probable AFM or another likely diagnosis). The cases for whom an alternative diagnosis was considered more likely showed no clear seasonality or relation with EV-D68/EV-A71 circulation, as shown in Figure 2.

## Discussion

We provide a minimal estimate of the incidence of AFM of 0.06/100,000 children/year (95% CI: −0.03 to 0.14) in the Netherlands from January 2014 through December 2019. The number of cases whom we finally classified as probable or definite AFM is low, but in line with the reported incidence of AFM in the literature [24,25].

EV-D68 was detected in five of 11 cases, classified as probable and definite AFM, while EV-A71 was not identified in any of the included patients. We found indications for a temporal relationship between the number of AFM patients and the number of EV-D68 positive samples identified in two different surveillance systems during the study period, which supports the previously established association between AFM and EV-D68 infection [2,5].

While AFM cases in Europe have been described in several case reports and case series, in particular during years of increased EV-D68 and EV-A71 circulation, our study is, to our knowledge, the first to provide incidence rates of AFM in a European country [13,16,26]. Surveillance in Europe has particularly focused on the identification of EV-D68, in which no link can be made with AFM. In surveillance studies, a yearly variation similar to that observed in this study was reported [10-12].

In the US, AFM incidence rates have been reported, mainly based on passive surveillance, which is prone to under-reporting and underestimation of the real incidence rate. In the general US population, the incidence rates were calculated as 0.01–0.07/100,000 inhabitants/year based on data from the US Centers for Disease Control and Prevention (CDC) from 2014 to 2020 with bi-annual peaks. Based on a short period of increased reporting in 2014, one study described an incidence of 0.32/100,000 population/year in individuals younger than 21 years [27]. From 2012 to 2015 incidence rates of 0.03–0.16/100.000 person-years in California in both children and adults were reported, with a clear temporal variation [25]. A retrospective cohort study in northern California reported higher incidence rates of 0.30–1.43/100,000 person-years in children between 1 and 18 years of age, with most cases reported in 2014 and 2016 [24]. Although all of these studies were performed before the introduction of the current AFM classification, diagnosis was also primarily based on the combination of acute flaccid limb weakness and spinal cord lesions largely restricted to the spinal cord grey matter. Similar to our findings, the temporal variation seemed to be connected to the circulation of different EVs, in particular EV-D68.

Our study has some limitations, including factors that may have led to an underestimation of the true incidence rate of AFM. First, patients with mild symptoms may not be referred to a paediatric neurologist, which may have led to a selection bias towards more severe cases. The full range of the clinical phenotype of AFM, possibly including milder cases, may only be revealed by large prospective cohort studies in both university and community hospitals.

Second, for the identification of patients we had to use diagnostic codes not specific for AFM, since the ICD-code for AFM has only been introduced in 2021. Some AFM cases are inevitably missed by this approach, as was confirmed by the identification of two suspected cases not included in the initial search.

Third, correct classification according to published AFM diagnostic criteria depends largely on additional investigations, in particular MRI. Cases with unrecognised or absent MRI abnormalities or in whom an MRI study was not performed are generally classified in a group with lower diagnostic certainty. On the other hand, cases of transverse myelitis may be unjustly classified as AFM as differentiation can be difficult based on current criteria. In particular, the clinical presentation of myelitis in the context of MOG-associated disease may be similar with AFP of the limbs and predominant grey matter abnormalities of the spinal cord on MRI [28].

Last, elucidating the temporal relationship between AFM cases and EV-D68/EV-A71 may be limited by suboptimal sensitivity of the surveillance systems at the beginning of an EV season. This might explain that AFM cases already occurred before increased EV-D68/EV-A71 detections were noted by the EV surveillance.

Despite its rarity and the lack of therapeutic options in the acute phase of the disease, the impact of AFM on affected children, which frequently results in severe residual deficits determines the urgency of monitoring this disease [29]. Insight in the epidemiology of AFM is important not only for the estimation of the burden of this disease, but also for better understanding of the causal relationship with viruses such as EV-D68 and EV-A71.

Increased awareness of AFM among clinicians will hopefully lead to its improved and early recognition, the relevance of which is shown in this study by the identification of several AFM cases (i.e. six of 11 in total) who were initially diagnosed with another neurological disorder, such as transverse myelitis. The relevance of awareness is illustrated by the identification of several cases in the Netherlands in the autumn of 2021. This is presumably related to an upsurge of EV-D68 across Europe that was identified to be higher than in previous years [30].

Appropriate viral diagnostics were not always performed in AFM cases in this study but are important to support the diagnosis and its relation with EV infections [31]. More intense collaboration between clinicians and virologists/microbiologists may help to ensure the performance of timely and adequate diagnostic tests, improving diagnostic accuracy as well as virus detection and identification. However, despite the importance of relating the incidence of AFM to EV epidemiology, surveillance solely based on EV infection would not be sufficient as an EV is not detected in all cases, both in our and prior studies.

Apart from adequate identification, registration of new AFM cases will be necessary to keep track of the incidence and determine the burden of disease and healthcare impact. Additionally, setting up national centres of expertise for spreading knowledge and information, for consultation and registration of AFM patients is recommended. The recently emerged European non-polio EV network (ENPEN) might provide a good structure to facilitate AFM case registration in Europe [9].

## Conclusion

AFM is a rare disease, but with significant impact on individual patients worldwide. Our minimum estimate of 0.06/100,000 children/year from 2014 through 2019 in the Netherlands is in line with previously reported incidence values from other countries. Our findings support the association between EV-D68 infection and AFM and the importance of adequate and timely virological testing. Identification of new cases may be improved by stronger cooperation between clinicians and virologists/microbiologists, preferably based on specific guidelines.

## Supplementary Data

220000 Supplementary Material
