# Supplementary material for: Epidemiology of acute flaccid myelitis in children in the Netherlands, 2014 to 2019
Source: Euro Surveill. 2022 Oct 20;27(42):2200157. doi: 10.2807/1560-7917.ES.2022.27.42.2200157 (PMC9585879; doi:10.2807/1560-7917.ES.2022.27.42.2200157)

This supplementary material is hosted by Eurosurveillance as supporting information alongside the article 'Epidemiology of acute flaccid myelitis in children in the Netherlands, 2014 to 2019', on behalf of the authors, who remain responsible for the accuracy and appropriateness of the content. The same standards for ethics, copyright, attributions and permissions as for the article apply. Supplements are not edited by Eurosurveillance and the journal is not responsible for the maintenance of any links or email addresses provided therein."

## DBC- and ICD-codes included in the search of this study

DBC ('Diagnose Behandel Combinatie')[1], translated from Dutch;

0330 – Neurology, Code 0191, Specific neurological infections

0330 – Neurology, Code 0199, Other neurological infections

0330 – Neurology, Code 0521, Anterior horn disorders

0330 – Neurology, Code 0542, Spinal cord disorders, not further specified

0330 – Neurology, Code 0811, Polyneuropathy infectious (GBS/CIDP)

0316 – Paediatrics, Code 3504, Guillain-Barré syndrome

0316 – Paediatrics, Code 3511, Meningitis/ encephalitis (CNS-infection)

ICD-10:

A86 – Unspecified viral encephalitis

G04.8 – Other encephalitis, myelitis and encephalomyelitis

G04.9 – Encephalitis, myelitis and encephalomyelitis, unspecified

G12.2 – Motor neuron disease

G37.3 - Acute transverse myelitis in demyelinating disease of the central nervous system

G61.0 – Guillain-Barré syndrome

[1] DBC-codes are based on a combination of diagnosis and treatment, which were introduced in the Netherlands in 2005.

**Supplementary Table S1:** Yearly incidence rates based on the number of cases classified as probable or definite acute flaccid myelitis (AFM). 95% CI: 95 percent confidence interval.

| **Year** | Number of AFM cases (definite and probable | Total population under 18 years (x $10^6$)[1] | Estimated population under 18 years in the area not covered (x $10^6$)[2] | Population under 18 years in covered area (x $10^6$)[3] | Incidence rate (/100.000 children) | Lower limit 95% CI | Upper limit 95% CI |
|---|---|---|---|---|---|---|---|
| 2014 | 1 | 3.44 | 0.20 | 3.24 | 0.03 | 0.15 | -0.02 |
| 2015 | 0 | 3.43 | 0.20 | 3.23 | 0 | 0 | 0 |
| 2016 | 4 | 3.44 | 0.20 | 3.24 | 0.12 | 0.24 | 0.00 |
| 2017 | 0 | 3.4 | 0.20 | 3.20 | 0 | 0 | 0 |
| 2018 | 3 | 3.38 | 0.19 | 3.19 | 0.09 | 0.29 | 0.02 |
| 2019 | 3 | 3.36 | 0.19 | 3.17 | 0.09 | 0.20 | -0.01 |
| **Mean** | 1.8 | 3.40 | 0.20 | 3.21 | 0.06 | 0.17 | -0.02 |

[1]Total number of children in the Netherlands according to population numbers of Statistics Netherlands (CBS).

[2] Estimated number of children in the referral region of the university hospital not participating in this study (area indicated in supplementary figure 2)

[3] Number of children after subtraction of the estimate number of children in the referral region of the university hospital not participating in this study

**Supplementary Table S2**: Month of onset of the enterovirus positive cases in the different categories[1].

| **Year** | Month | Definite AFM | | Probable AFM | | Other diagnosis more probable | |
|---|---|---|---|---|---|---|---|
| | | EV[2] | EV-D68 | EV[2] | EV-D68 | EV[2] | EV-D68 |
| 2016 | July | 0 | 0 | 1 | 1 | 0 | 0 |
| | August | 2 | 1 | 0 | 0 | 0 | 0 |
| 2018 | August | 1 | 1 | 0 | 0 | 1 | 0 |
| | October | 1 | 0 | 0 | 0 | 0 | 0 |
| 2019 | April | 0 | 0 | 0 | 0 | 1 | 0 |
| | May | 0 | 0 | 0 | 0 | 1 | 0 |
| | December | 1 | 1 | 0 | 0 | 0 | 0 |

[1] The categories 'possible AFM' and 'uncertain' are not shown, because no enterovirus was found in these groups

[2] Total number of identified enteroviruses, including both EV-D68 and other subtypes.

**Supplementary Figure S1:** Map of the Netherlands, showing the estimated area covered by the participating university hospitals and the location of all participating hospitals in this study.

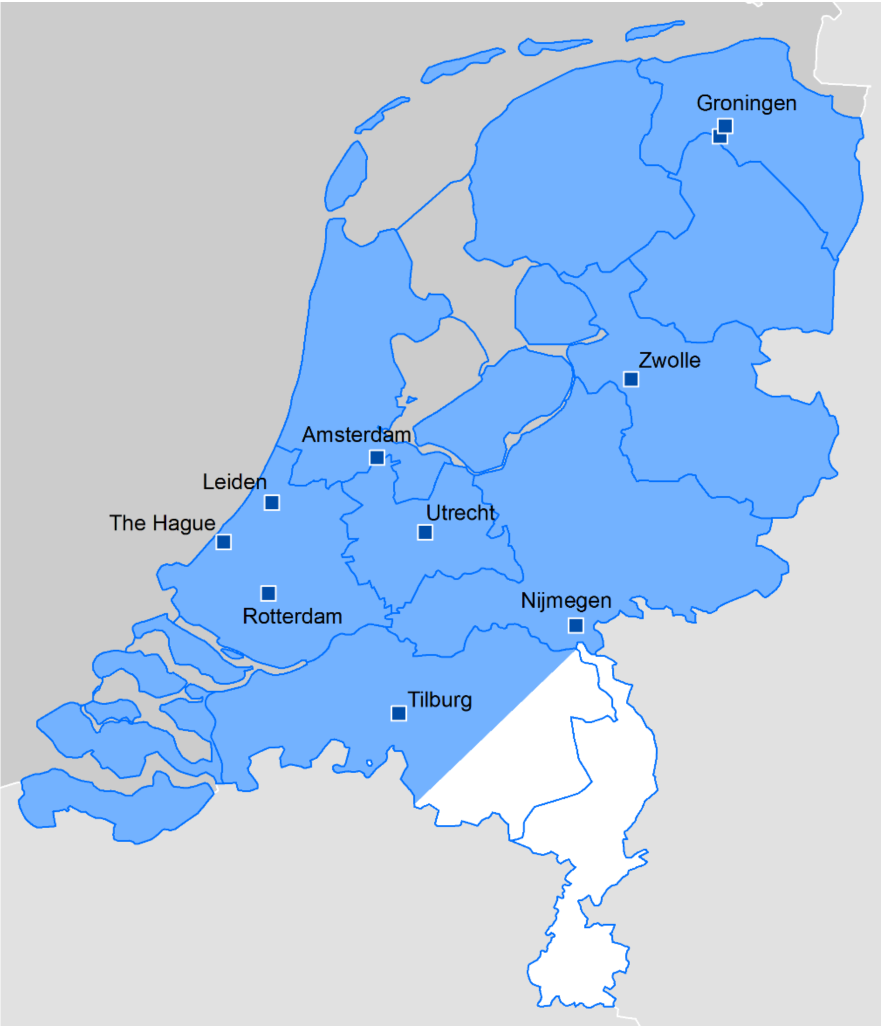

Supplement: Supplementary Material [file 2200157_SupplementaryMaterial.pdf]
